# Acquired Coagulopathy and Hemorrhage Secondary to Subcutaneous Heparin Prophylaxis

**DOI:** 10.1155/2018/9501863

**Published:** 2018-01-10

**Authors:** Maria Sunseri, Tania Ahuja, Tanya Wilcox, David Green

**Affiliations:** ^1^Department of Medicine, New York University Langone Health, 550 First Avenue, New York, NY 10016, USA; ^2^Department of Pharmacy, New York University Langone Health, 550 First Avenue, New York, NY, USA; ^3^Department of Medicine, New York University Langone Health, Division of Hematology, 550 First Avenue, New York, NY, USA

## Abstract

Unfractionated heparin and low-molecular-weight heparins are commonly used as thromboprophylaxis for hospitalized patients. Though generally considered safe at prophylactic doses, cases of catastrophic hemorrhage have been reported. The proposed mechanism involves bioaccumulation of heparin through saturation of the rapid-elimination pathway in its metabolism. We present an unusual case of an average-weight man with metastatic melanoma who suffered hemorrhage with syncope and end-organ damage while on prophylactic three times daily unfractionated heparin. Coagulation studies were consistent with heparin toxicity. Despite administration of protamine, the clearance of heparin was remarkably delayed, as demonstrated by serial coagulation studies. We review the suspected risk factors for heparin bioaccumulation and the emerging understanding of this unusual adverse event involving a nearly ubiquitous medication.

## 1. Background

Subcutaneous heparin is a common method of thromboprophylaxis used among heterogeneous populations of hospitalized patients for the prevention of venous thromboembolism (VTE) [1]. The benefit of VTE prophylaxis in medical and surgical inpatients has been widely demonstrated and is promoted by national guidelines [2], which use the available data to make graded recommendations for thromboprophylaxis based on an individual patient's risk of thrombosis or bleed. Further, organizations such as The Joint Commission have emphasized thromboprophylaxis as a key quality measure [2], such that many institutions are implementing computerized clinical decision support tools that prompt providers to order or withhold VTE prophylaxis for all patients at admission and at regular intervals throughout the hospitalization [2]. Patients with malignancy are known to suffer high rates of in-hospital morbidity and mortality from VTE [3, 4]. As such, available guidelines from sources such as the American Society for Clinical Oncology recommend routine thromboprophylaxis in cancer patients admitted for acute illness without apparent contraindication to anticoagulation [4]. Clinicians, in turn, are more likely to regularly order pharmacologic VTE prophylaxis in patients with known cancers.

Although there are tools available to assess thromboembolic risk, there is insufficient evidence to recommend which pharmacologic agent to use for VTE prophylaxis in hospitalized patients. Low-molecular-weight heparin may cause less bleeding complications compared to unfractionated heparin (UFH) and is easier to administer; however, its use is precluded by renal insufficiency and cost [5]. Heparin's widespread use as thromboprophylaxis is in part due to its relative safety profile at the doses effective in reducing risk of clinically significant VTE [1, 6–10]. However, clinically significant hemorrhage from heparin prophylaxis has been reported in the literature [11–14]. In fact, in hospitalized medical patients, prophylaxis with heparin is associated with an increase in all bleeding events (absolute increase, 9 events per 1000 persons treated) and a nonstatistically significant increase in major bleeding events (absolute increase, 1 event per 1000 persons treated) [15]. Elimination of UFH is related to two mechanisms; initially, heparin is cleared through the reticuloendothelial system by a saturable, rapid, zero-order process [16]. At high doses, this system saturates, and further elimination occurs renally via a first-order process that is slower and nonsaturable [16]. It is suspected that prolonged use in a high-risk patient may lead to saturation of the rapid-elimination pathway and bioaccumulation of heparin, resulting in iatrogenic coagulopathy and increased risk of major hemorrhage. Furthermore, the dose of UFH for prophylaxis varies from 5000 units twice daily (BID) to three times daily (TID) without a head-to-head comparison. A meta-analysis provided a comparison between BID and TID UFH for prophylaxis in general medical inpatients and found that rates of minor bleeding were similar between the two dosing regimens; however, major bleeding occurred more frequently with TID than with BID dosing (0.73 versus 0.33 events per 100 patient-days; *P* < 0.001) [17]. Although not well studied, in a subanalysis, data from the International Medical Prevention Registry on Venous Thromboembolism (IMPROVE) assessed in-hospital bleeding to identify risk factors on admission in acutely ill medical patients [18]. This analysis found independent factors associated with 14-day in-hospital bleeding to be active gastroduodenal ulcer, bleeding during the 3 months prior to admission, thrombocytopenia, advanced age, hepatic failure, renal failure, intensive care stay, presence of a central venous catheter, rheumatic disease, current cancer, and male sex [18]. In another study of elderly medical patients treated with prophylactic dosages of enoxaparin, the authors found renal impairment, defined as a creatinine clearance of <30 mL/min, and body weight < 50 kg to be associated with significantly higher antifactor Xa (anti-Xa) values [19]. There were no clinical conclusions drawn from this study regarding the safety of enoxaparin in elderly medical patients [19]. Given the paucity of data, clinicians must maintain a high index of suspicion for bleeding complications of pharmacologic thromboprophylaxis.

If heparin overdose is suspected clinically, the diagnosis can be supported by prolonged activated partial thromboplastin time (aPTT) or elevated anti-Xa assay. Prolonged thrombin time (TT) can help identify heparin effect, especially if a reptilase time (RT) is available. Reptilase time, like thrombin time, measures the conversion of fibrinogen to fibrin; however, it is not sensitive to the effects of heparin, and thus if normal can help localize the coagulopathy to a complication of heparin therapy [20].

Currently, there are no routine recommended monitoring parameters for low-dose heparin prophylaxis in hospitalized patients. In addition, without a head-to-head comparison, many clinicians consider dosing UFH TID for patients at highest risk for thromboembolism and BID for those at highest risk for bleeding [17]. Coagulation studies such as aPTT or anti-Xa may be useful for monitoring certain patients who are at increased risk of bioaccumulation and adverse outcome. We present a case of a patient on prolonged heparin thromboprophylaxis who experienced hemodynamically significant hemorrhage from probable bioaccumulation despite lacking the classic risk profile. Informed consent was obtained from the patient for publication of this case.

## 2. Case Presentation

A 68-year-old Colombian man who worked as an artist was diagnosed with stage IV metastatic melanoma two years prior to presentation after finding a lump in his groin. His past medical history was significant for well-controlled hypertension, hyperlipidemia, insulin-dependent type 2 diabetes, stage III chronic kidney disease, prostate cancer, and gout; he was also born with a single kidney. He experienced partial regression of his melanoma with pembrolizumab but developed acute interstitial nephritis; thus, therapy was suspended and the patient was treated with methylprednisolone followed by high-dose prednisone and furosemide. He then underwent gamma knife resection of a temporal lobe brain metastasis; his recovery was complicated by bilateral lower extremity deep vein thrombosis (DVT) treated with inferior vena cava filter; therapeutic anticoagulation was deferred in the setting of recent brain surgery. One week after discharge to an acute rehabilitation facility, he returned to the hospital complaining of urinary retention.

On presentation, the patient was found to have an enterococcal urinary tract infection and an acute kidney injury. He was started on ciprofloxacin for the infection; high-dose prednisone was resumed in light of his recent interstitial nephritis. His presentation was also notable for new acute occlusive DVTs in the bilateral lower extremities. Again, therapeutic anticoagulation was deferred given bleeding risk in the postoperative period. He was given prophylactically dosed enoxaparin, which was changed to heparin on hospital day 8 given worsening renal function. Heparin was administered subcutaneously and dosed TID as per institutional weight-based dosing protocols. Staging imaging revealed progression of his disease with diffuse lymphadenopathy and peritoneal carcinomatosis. Given worsening clinical status, it was decided to start vemurafenib, a B-type Raf (BRAF) inhibitor, and cobimetinib, a mitogen-activated protein kinase (MEK) inhibitor, at reduced doses.

**Figure 1:**
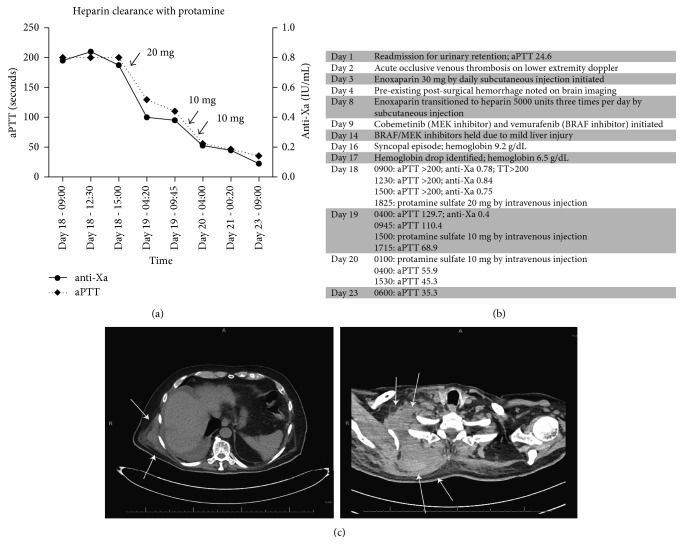
(a) Timeline of heparin clearance with protamine administration. Left *y*-axis: aPTT; right *y*-axis: anti-Xa. Arrows demarcate timing and dose of protamine administration. aPTT: activated partial thromboplastin time; reference range 27–38 seconds. Anti-Xa: antifactor Xa activity; reference 0 or <0.3 IU/mL for prophylactic dosing; reference range 0.3–0.7 for therapeutic anticoagulation. (b) Clinical timeline. Activated partial thromboplastin time and thrombin time measured in seconds. Anti-Xa measured in IU/mL. (c) Representative axial images from the patient's chest CT demonstrating multifocal soft tissue hematomas (arrows).

On day 16 of hospitalization, the patient became unresponsive while sitting on the edge of his bed. On regaining consciousness, his blood pressure was 80/50 with heart rates ranging 120–130. He reported feeling dizzy and light headed. His vital signs quickly returned to baseline with symptomatic resolution. Physical exam was notable for right-sided supraclavicular fullness and tenderness to palpation, pain with passive range of motion of the right shoulder, and diffuse abdominal ecchymoses at sites of prior heparin injections. Immediate labs demonstrated elevated troponin and lactate. The following morning an acute drop in hemoglobin was noted, from 9.2 g/dL to 6.5 g/dL over 24 hours. Computed tomography (CT) of the chest revealed an expansive subcutaneous hematoma involving the posterior chest wall and right supraclavicular region (Figure 1). The hematoma appeared to be associated with sites of previously unknown metastatic lesions. Coagulation studies demonstrated markedly elevated aPTT (>200 s), thrombin time (>200 s), and anti-Xa (0.83 IU/mL). The constellation of lab findings indicated heparin toxicity.

The patient was supported with transfusions and reversed with several doses of intravenous (IV) protamine sulfate. The aPTT decreased after 20 mg of protamine from >200 s to 129.7 s and then 110.4 s. Two additional 10 mg doses were given with decrease to 68.9 s and 55.9 s, respectively. The aPTT normalized to 35.3 s the following day with no additional doses (Figure 1). Of note, despite the administration of protamine, the heparin clearance was remarkably delayed (Figure 1).

The patient recovered from his acute bleed, heparin was held indefinitely, and he did not have recurrence of his coagulopathy. Ultimately, however, his liver function worsened with the chemotherapy, requiring drug changes and further dose reductions. There was no reduction in his tumor burden despite trials of multiple agents, and his kidney function again declined. The patient was eventually transitioned to inpatient hospice.

## 3. Diagnostic Assessment

Measurement of aPTT, anti-Xa, and TT were made by standard specimen analysis in the clinical laboratory. Results may be spurious if the sample is drawn from a heparinized line or via traumatic venipuncture. In this case, each specimen was drawn from a peripheral indwelling central catheter; prior to collection, the line was appropriately flushed with normal saline, and the first 10 mL of collected blood was discarded.

## 4. Conclusions

This case describes an unusual adverse event involving a nearly ubiquitous medication. This patient's known malignancy and history of recurrent DVT made him a strong candidate for VTE prophylaxis. Although his recent brain surgery and metastatic melanoma precluded him from therapeutic anticoagulation due to risk of hemorrhage, he had no active bleeding and no history of significant bleed, suggesting minimal or no contraindication to low-dose TID heparin as thromboprophylaxis. The suggested risk factors for heparin bioaccumulation do not fit this patient, with exception of male sex and cancer. He was of average body weight, and his renal failure was acute with his creatinine clearance improving at the time of heparin administration. Despite this, he suffered hemodynamically significant hemorrhage with laboratory markers of end-organ damage that took several days to clear. The bleed appears to have originated from metastatic lesions visualized on his chest imaging. This case sheds further light on the emerging understanding of heparin bioaccumulation, suggesting that there may be as-yet unidentified factors placing certain patients at risk. Since heparin has been shown to be depolymerized by endothelial cell lysosomal enzymes [21], we hypothesize that the effects of this patient's malignancy or the BRAF or MEK inhibitors may have contributed to endothelial dysfunction and delayed heparin clearance. Reporting patients that suffer this outcome is important to elucidating the pathogenesis of bioaccumulation and eventually identifying parameters for patients that may require closer monitoring with targeted coagulation studies during prolonged use of heparin thromboprophylaxis.

## Abbreviations

UFH: Unfractionated heparin
anti-Xa: Antifactor Xa activity
aPTT: Activated partial thromboplastin time
TT: Thrombin time
RT: Reptilase time
BID: Twice daily
TID: Three times daily
VTE: Venous thromboembolism
DVT: Deep vein thrombosis
BRAF: B-type Raf
MEK: Mitogen-activated protein kinase
CT: Computed tomography
IV: Intravenous.
